# Supplementary material for: The Immune System Response to 15-kDa Barley Protein: A Mouse Model Study
Source: Nutrients. 2022 Oct 18;14(20):4371. doi: 10.3390/nu14204371 (PMC9611736; doi:10.3390/nu14204371)
Supplement: Supplementary file 1 [file nutrients-14-04371-s001.zip › 0_Supplementary Figure S1.pdf]

The Immune System Response to 15-kDa Barley Protein: A Mouse Model Study

Barbara Wróblewska [1], Ewa Kubicka [1], Ewelina Semenowicz [1], Anna Ogrodowczyk [1], Anita Mikołajczyk [2] and Dagmara Złotkowska [1,*]

[1] Institute of Animal Reproduction and Food Research, Polish Academy of Science Department of Food Immunology and Microbiology, Tuwima Str. 10, 10-748 Olsztyn, Poland

[2] Department of Public Health, Faculty of Health Sciences, Collegium Medicum, University of Warmia and Mazury, 10-082 Olsztyn, Poland

* Correspondence: d.zlotkowska@pan.olsztyn.pl

2. Materials and Methods

2.2 Beer

We bought the beer Kormoran Brewery Pilsener (BK) at the local market. According to the manufacturer, the beer was pilsner type, light, 12.5% extract (w), and 5.2% alcohol (v). After lyophilization, solution at 100⊚g/mL concentration was added to mice lymphocyte cultures as a stimulation agent.

2.5 Sodium Dodecyl Sulfate–Polyacrylamide Gel Electrophoresis (SDS-PAGE)

Lyophilized beer (BK; at a concentration of 1 mg/mL) was subjected to SDS- PAGE according to a standard protocol used in the laboratory [17]. Briefly, proteins were separated on 12.5% gels under a constant current of 30 mA using a Mini-PROTEAN system (Bio-Rad Laboratories, Inc., Warsaw, Poland). Gels were stained with 0.1% Coomassie Brilliant Blue R-250 (Sigma, Poland). We used Precision Plus Protein™ Standards (MWS) in the range of 10–250 kDa (Bio-Rad, USA) to estimate proteins' molecular weight profile.

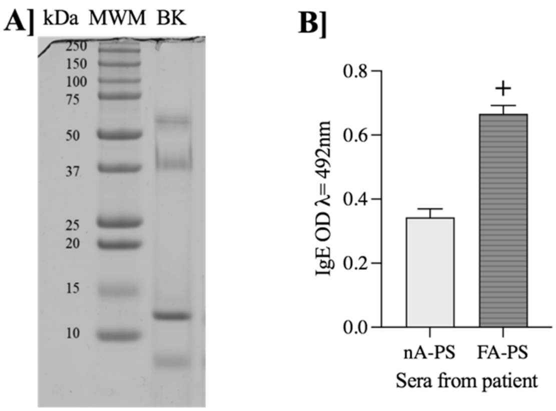

Supplementary Figure S1 SDS-PAGE profile (A) and IgE immunoreactivity (B) of lyophilized beer. Abbreviation: MWM – molecular weight marker; BK – Kormoran Brewery Pilsener Beer liophylisate; nA-PS – sera from patients non-allergic to food; FA-PS-sera from patients allergic to food. Mean OD value ± SD was considered a positive response (assigned with +) if it exceeded the mean of negative controls (nA-PS) by more than three standard deviations.
