# Supplementary material for: The Immune System Response to 15-kDa Barley Protein: A Mouse Model Study
Source: Nutrients. 2022 Oct 18;14(20):4371. doi: 10.3390/nu14204371 (PMC9611736; doi:10.3390/nu14204371)
Supplement: Supplementary file 1 [file nutrients-14-04371-s001.zip › 0_Supplementary Table S1.pdf]

The Immune System Response to 15-kDa Barley Protein: A Mouse Model Study

Barbara Wróblewska [1], Ewa Kubicka [1], Ewelina Semenowicz [1], Anna Ogrodowczyk [1], Anita Mikołajczyk [2] and Dagmara Złotkowska [1,*]

[1] Institute of Animal Reproduction and Food Research, Polish Academy of Science Department of Food Immunology and Microbiology, Tuwima Str. 10, 10-748 Olsztyn, Poland

[2] Department of Public Health, Faculty of Health Sciences, Collegium Medicum, University of Warmia and Mazury, 10-082 Olsztyn, Poland

* Correspondence: d.zlotkowska@pan.olsztyn.pl

Table S1. In silico analysis of barley proteins immunogenicity according to strong binding to MHC II alleles.

No.	UniProt [a]	Protein Name	MW (fragments) [kDa]	Strong binders to MHC alleles			Number of peptides		Allergenicity [d]
				DRB1	DQ	DP	IFN Inducers [b]	IL4 Inducers [c]	
1	F2EKW4	Predicted protein (Calcium-binding Proteins)	8.622	10	5	7	15	48	*In silico* modeled allergen (10234; Hor v 7)
2	A8V3P3	Chymotrypsin inhibitor-2	9.381	12	5	7	22	38	*In silico* modeled allergen (10785; Hor v 39)
3	P07597	Non-specific lipid-transfer protein 1 (LTP1)	12.301	11	5	7	38	54	Confirmed allergen also for barley (1059; Tri a 14)
4	F2EC90	Predicted protein (Expansin-like CBD domain-containing protein)	13.163	10	5	7	30	55	Confirmed allergen (1321; Hor v 2)
5	F2CT70	Profilin	14.142	9	5	7	14	94	Confirmed allergen (1406; Hor v 12)
6	F2EE63	Acidic protein (Beta-hordothionin)	14.573	12	5	7	19	85	Confirmed allergen (11368; Hor v 37)
7	C3VX00	Dimeric alpha-amylase inhibitor	15.17	11	5	7	6	79	*In silico* modeled allergen (9186; Hor v 28)
8	1208404A (No. NCBI)	Alpha-amylase/trypsin inhibitor pUP 13	15.17	12	5	7	17	109	Non-described allergen for barley (but allergenicity described for pUP13 protein-17kDa alpha-amylase/trypsin inhibitor 1)

9	P16159	Alpha-amylase/trypsin inhibitor CM16	15.782	9	5	7	8	61	Confirmed allergen also for barley (8777; Tri a 40)
10	P16968	Alpha-amylase inhibitor BMAI-1	15.816	12	5	7	30	77	Confirmed allergen (3328; Hor v 15)
11	P01086	Trypsin inhibitor CMe	16.136	9	5	7	15	80	Confirmed allergen (8779; Hor v BTI)
12	P13691	Alpha-amylase inhibitor BDAI-1	16.429	11	5	7	22	81	Confirmed allergen (8778; Hor v BDAI)
13	Q9M4E3	Hordoindoline-A	16.494	10	5	7	34	102	Non-described allergen for barley (*In silico* modeled potential allergen)
14	Q9LEH8	Hordoindoline-B2	16.78	12	5	7	35	113	Non-described allergen for barley (*In silico* modeled potential allergen)
15	F2EHP3 (A0A0D9YNR2)	Polygalacturonase	45.751 (17.806)	12	5	7	26	335	Confirmed allergen (2526; Hor v 13)
16	P17314	Alpha-amylase/trypsin inhibitor CM3	18.221	12	5	7	37	120	Confirmed allergen also for barley (1051; Tri a 30)
17	O04828 (Q39995)	Pollen_allerg_2 domain-containing protein	33.148 (18.371)	12	5	7	63	124	Confirmed allergen (422; Hor v 5)
18	C3W8L9 (F6KU73)	Starch synthase, chloroplastic/amyloplastic	66.280 (18.998)	12	5	7	31	114	*In silico* modeled allergen (11011; Hor v GBSS_I)
19	O23978 (A0A453BCA6)	Beta-Amylases	57.308 (19.596)	11	5	7	36	130	Confirmed allergen (420; Hor v 17)
20	A0A3B6JDX7 (Q70JP9)	Predicted protein-(Putative major allergen Phl p 5-Fragment)	33.959 (23.949)	12	5	7	55	168	Confirmed allergen also for barley (745; Tri a 5)
21	P52572	1-Cys peroxiredoxin	23.963	9	5	7	21	160	Confirmed allergen (9882; Hor v 32)
22	P06293 (A0A287SR04)	Serpin-Z4 (SERPIN domain-containing protein)	43.276 (28.076)	12	5	7	60	60	Confirmed allergen (951; Hor v 33)
23	P80198	Gamma-hordein-3 (Gliadin)	33.189	11	5	7	66	228	Confirmed allergen (3331; Hor v 20)

a) ACCESSION (No. To UniProt or reference database).
b) tracked with use http://crdd.osdd.net/raghava/il4pred/
c) tracked with use https://webs.iiitd.edu.in/raghava/ifnepitope/
d) Allergome database code, name of alleregene, and information if it is in vivo proven or in silico modeled allergen - analysis based on Alergpred tool and Allergenonline V.21)
